# Polyurethane-Solidified Ballast Under Unconfined and Confined Conditions: Laboratory Load Testing and Mesoscopic Analysis

**DOI:** 10.3390/ma19091863

**Published:** 2026-05-01

**Authors:** Wei Chen, Shuojun Chen, Shang Luo, Yushuo Zhang, Weidong Wang, Qiang Yuan

**Affiliations:** 1 School of Civil Engineering, Central South University, Changsha 410075, China; chenwei.csu@foxmail.com (W.C.);; 2 MOE Key Laboratory of Engineering Structures of Heavy-Haul Railway, Central South University, Changsha 410075, China

**Keywords:** polyurethane solidified ballast, ballasted track bed, mechanical behavior, laboratory test, discrete element analysis

## Abstract

**Highlights:**

- Comparative mechanical response of polyurethane-solidified ballast (PSB) under confined versus unconfined states.
- The PSB shows superior energy dissipation and damping capacity compared to traditional ballast.
- DEM simulations reveal particle-scale load transfer and bond evolution.

**Abstract:**

The prefabricated polyurethane-solidified track bed (PPSTB) combines the adjustability of ballasted tracks with the low maintenance requirements of slab tracks, offering a promising solution for railway sections on deformable foundations. This study investigates the interaction and mechanical behaviors of the polyurethane-solidified ballast (PSB) modules and bulk ballast under laboratory loading. A series of unconfined uniaxial tests, confined ballast box tests, and cyclic loading tests were conducted, complemented by discrete element method (DEM) simulations to analyze mesoscopic particle evolution. Under monotonic compression, the stress–strain curve exhibits three distinct stages with an average elastic modulus of 19.66 MPa, where the central aggregate framework acts as the primary load-bearing structure. Confinement increases the modulus by 33.57% and yields a nearly linear stress–strain relationship, attributed to a more compact and uniform contact distribution. Furthermore, under cyclic loading, the PSB shows enhanced energy dissipation and deformation resistance compared to conventional ballast. These findings provide a theoretical basis for the structural design and long-term performance assessment of the PPSTB.

## 1. Introduction

The railway track bed constitutes the foundational structure of the railway track system, serving to bear and transmitting the cyclic loads generated by passing trains. Ballasted track beds are commonly employed in railway projects within complex geological environments, owing to their cost-effectiveness, ease of repair, and adjustability. Nevertheless, inherent problems such as ballast breakage and track deformation increase railway maintenance costs and even elevate the risk of track instability [1,2,3,4,5]. The polyurethane-solidified track bed (PSTB) offers a novel solution to resolve these challenges in railway engineering. This type of track bed combines the superior stability of ballastless track beds with the high adjustability of ballasted track beds [6,7]. Its installation has been implemented along numerous railway lines throughout China, including the subgrade–bridge transition zone and tunnel crossing active fault zone [8,9,10].

**Figure 1:**
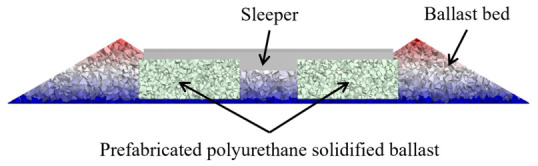
Cross-section of the PPSTB.

The PSTB technology initially employed on-site pouring construction, in which polyurethane material is injected into the ballasted track bed [11,12,13]. Upon foaming and curing, the material fills the voids between ballast particles, forming a rigid bonding structure [14]. This effectively restricts ballast displacement and force transmission under dynamic train loads, thereby mitigating residual deformation and ballast deterioration. However, the rigid bonding compromises the ballast bed’s inherent elasticity and poses significant challenges for subsequent maintenance and replacement. The in situ casting process faces stringent construction conditions and extended timelines [15]. In contrast, the prefabricated polyurethane-solidified track bed (PPSTB) employs a factory-precast, in situ assembly approach using pre-foamed polyurethane, as illustrated in Figure 1. The prefabricated polyurethane-solidified ballast modules (denoted by light-colored particles) are hoisted to the construction site for assembly. The bulk ballast is thereafter backfilled to form the complete track bed. This track bed offers advantages such as strong energy absorption capability, environmental adaptability, and simplified construction procedures [16,17,18].

During service, the polyurethane-solidified ballast (PSB) modules within the PPSTB primarily bear the dynamic cyclic loads transmitted from the sleepers, operating under a complex stress state dominated by vertical loads [19]. Consequently, researchers first investigated the intrinsic mechanical properties of the PSB through various mechanical tests. Keene et al. [20] investigated the changes in mechanical properties following polyurethane–ballast bonding through uniaxial compression and flexural tests under cyclic loading. Lee et al. [21] conducted large-scale triaxial tests on polyurethane-mixed coarse aggregates and quantitatively investigated the relationship between stiffness and strength of the PSB and polyurethane content. Gundavaram et al. [22] conducted large-scale direct shear tests on Elastan-stabilized ballast, revealing that polyurethane-stabilized ballast exhibits higher shear strength than geogrid-reinforced variants. Zhang et al. [23] designed a series of tensile, compressive, and shear tests to investigate the mechanical characteristics of the bond interface between polyurethane and ballast. Prasad and Hussaini [24] determined the optimum polyurethane content that balances both the service performance and cost-effectiveness of ballast through large-scale direct shear and cyclic loading tests. Current experimental research predominantly focuses on the mechanical properties of the PSB itself. Although the PSB modules constitute the primary load-bearing component in the PPSTB, the influence of backfilled bulk ballast cannot be disregarded. The bulk ballast provides confining pressure to the PSB modules, which not only enhances the load-bearing capacity but also offers lateral resistance. Consequently, it is necessary to investigate the interaction between the PSB and bulk ballast and their comprehensive mechanical properties, establishing a correlation between the block test and actual in-service performance.

To obtain mechanical characteristics and performance parameters more closely aligned with actual service conditions, researchers have progressively undertaken field testing incorporating track structures. Huang et al. [25] established a comprehensive empirical formula system for predicting the lifting behaviors of slab tracks using foamed polyurethane grouting through full-scale track slab lifting tests. Xiong et al. [26] conducted hammer excitation tests to compare the dynamic performance of the PSTB and traditional ballasted track bed under high-speed conditions. Gundavaram and Hussaini [27] employed a large-scale track process simulation test apparatus to investigate the impact of different polyurethane treatment zones on track performance. However, the mechanical behavior of the track bed under train loading is a long-term evolutionary process. Current understanding of the long-term evolution of the PPSTB remains far less comprehensive than that of traditional ballasted track beds. Therefore, cyclic loading tests on the combined PSB and bulk ballast must also be conducted to obtain the mechanical response and dynamic characteristics of the PPSTB under long-term cyclic loading.

While some field-scale or track-scale studies have evaluated the global performance of polyurethane-reinforced track beds, they provide limited information on the mesoscopic mechanisms governing stiffness evolution, load transfer, and bond degradation within the PSB. As a result, the relationship between macroscopic response and internal particle-scale evolution remains insufficiently understood. The interactions between ballast particles are fundamental to the service performance of the track beds. In the traditional ballasted track bed, the load-bearing capacity and stability are primarily governed by the contact and interlocking of individual ballasts [28]. In contrast, the PSB derives its mechanical integrity from the adhesive properties of the polyurethane binder. Understanding the evolutionary process of the PSB at the particle scale is therefore crucial. Given that experimental methods are often limited by observational constraints at the micro-scale, discrete element method (DEM) simulations offer an effective alternative for capturing particle-level behavior. For instance, Ling et al. employed DEM modeling combined with laboratory tests to investigate shear resistance [29], dynamic characteristics [30], and energy evolution [31] of the polyurethane-mixed ballast from a macro–micro coupling perspective. Similarly, Xu et al. [32] established DEM models for various ballast gradations and proposed a parameterization methodology for the resilient PSB. Building on these advancements, the previous work by Zhang et al. [33] regarding the PSB mechanical properties and parameter sensitivity provides a robust foundation for the mesoscopic analysis model employed in this study.

This study investigates the mechanical behavior of PSB under both unconfined and confined conditions through laboratory tests and DEM analysis. The main objectives are (1) to compare the stress–strain response and deformation characteristics of PSB under different boundary conditions; (2) to evaluate its cyclic deformation, energy dissipation, and damping behavior in comparison with pure ballast; and (3) to reveal the mesoscopic mechanisms of contact-force redistribution and bond evolution by means of DEM simulation. The results are expected to improve the understanding of the coupled role of the bonded ballast skeleton and surrounding bulk ballast and to provide support for the design and application of prefabricated polyurethane-solidified track beds.

## 2. Laboratory Tests of Polyurethane-Solidified Ballast

### 2.1. Preparation of the PSB Specimens

#### 2.1.1. Bulk Ballast

According to the requirements for the gradation of premium-grade ballast in China, the minimum particle size is set at 16 mm and the maximum at 63 mm. Relevant studies indicate that the specimen size needs to be ≥350 mm to effectively eliminate the influence of size effects on test results [34,35]. However, the preparation process of such large specimens is not only complex but also involves substantial fabrication costs and time investment. In view of this, the scaled-down test, as an efficient alternative solution, has significant advantages in reducing test costs and improving efficiency. Yao et al. [36] confirmed, through comparative tests, that the results of the ballast scaled-down test are similar to those of the full-scale test, concluding that parameters obtained from scaled tests can be applied to engineering design. In addition, the pore size after polyurethane foaming is approximately 200 μm [37], significantly smaller than the ballast dimensions. Therefore, the scaled-down test will not affect the mechanical behavior of polyurethane. In this study, 150 mm cubic specimens were adopted as a practical laboratory-scale representation. This choice was based on previous studies showing that scaled ballast tests can reproduce the main mechanical response trends of larger specimens, while greatly reducing the specimen preparation cost and testing difficulty. In addition, relevant research has indicated that the influence of boundary effects on test results can only be neglected when the container size exceeds eight times the average particle size [38]. The ballast particle size range used in this study is 9.5 mm to 26.5 mm, with d_50_ = 18 mm, which is less than eight times the side length of the mold. Therefore, the boundary effects of the mold can be reduced.

**Figure 2:**
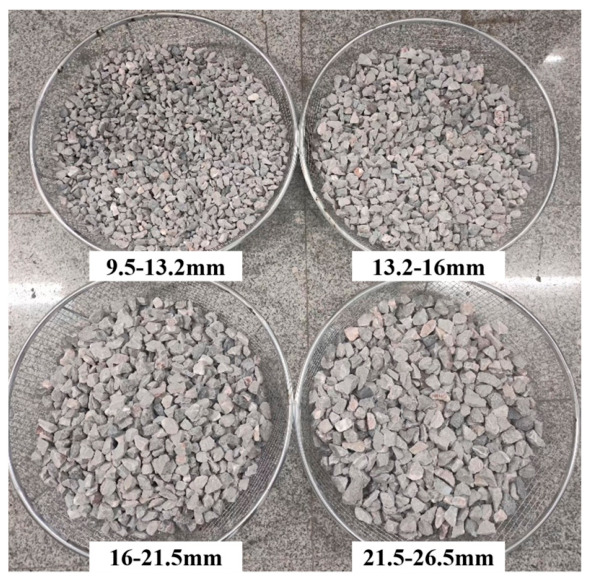
The four types of aggregate with different particle size ranges obtained after sieving.

Ballast particles used in the tests were provided by Hunan Junjia Road Surface Materials Co., Ltd. (Changsha, China). To meet the grading requirements, square sieves were used to grade ballast particles, yielding four distinct particle size ranges (26.5–21.5 mm, 21.5–16 mm, 16–13.2 mm, and 13.2–9.5 mm). The gradation of ballast particles after scaling is detailed in Table 1, with the sieving process illustrated in Figure 2. As the surface cleanliness of ballast particles directly affects the strength of the polyurethane bonding interface, the sieved ballast particles were placed in a water tank for rinsing until no further turbid substances were released. The ballast particles were dried to a visually dry surface condition before mixing.

#### 2.1.2. Polyurethane Materials

Polyurethane curing materials are synthesized through the polymerization and cross-linking reaction of isocyanates and polyols under catalytic action. The employed isocyanate and polyol raw materials are provided by Shanghai Huafon Material Technology Institute, blended at a mass ratio of 54:100. After thorough reaction, a resilient cured body with uniform and fine pores, without any cracks or holes, was formed. Through microscopic characterization, it was confirmed that the pore structure was intact and free of any micro-structural defects, exhibiting an excellent elastic deformation capacity and fracture elongation properties. The foamed density of the polyurethane curing material used in this study is 140 kg/m^3^ [39].

#### 2.1.3. Specimen Preparation Method

The specimens were fabricated using a cubic mold with a size of 150 mm. Polyurethane materials exhibit high viscosity during curing and generate intense heat release. To address potential mold adhesion issues, we coated the inner walls of the molds with lubricants, followed by covering them with an ultra-thin plastic film. This step not only enables non-destructive demolding, ensuring the integrity of the specimen’s surface, but also effectively prevents the penetration of lubricants.

Each specimen contains 5.57 kg of ballast in total, with the mass distribution across different particle size ranges detailed in Table 2. The amount of polyurethane used per specimen is 450 g.

**Figure 3:**
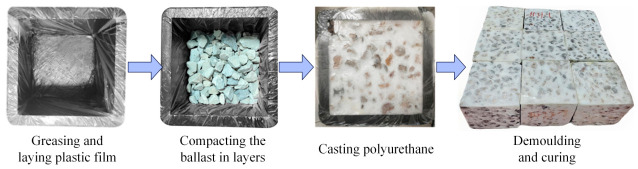
The production process and final product of the PSB specimens.

The procedure for preparing the PSB specimens is as follows (also illustrated in Figure 3):

(1) Weigh the ballast and polyurethane raw materials.

(2) Load the ballast into the mold in three portions. Vibrate for 30 s to ensure complete mixing. After thorough compaction, fix the cover plate to the top of the mold using the fastening bolts.

(3) Pour the polyurethane through the pre-drilled pouring hole in the cover plate.

(4) Maintain curing temperature between 20 and 30 °C. Testing may commence after 7 days of curing.

### 2.2. Laboratory Test Procedure

#### 2.2.1. Unconfined Tests

The unconfined compression test can reveal the compressive characteristics of the specimen. In this study, tests were conducted on three specimens (designated as C1, C2, and C3, respectively) produced by the aforementioned method from the same batch. The specimens were first carried on a load–unload test and, subsequently, a monotonic compression test until failure.

(1) Load–unload test: The vertical loading rate was 2 mm/min, and the loading amplitude was 10 kN. At this amplitude, the specimen remains in the elastic state. The stress–strain curve of each single load–unload process was recorded.

(2) Monotonic compression test: The vertical loading rate was 2 mm/min until the vertical strain reached 30%, at which point the specimen was deemed to have lost its load-bearing capacity. The stress–strain curve during this monotonic loading process and the final shape of the specimen were recorded.

**Figure 4:**
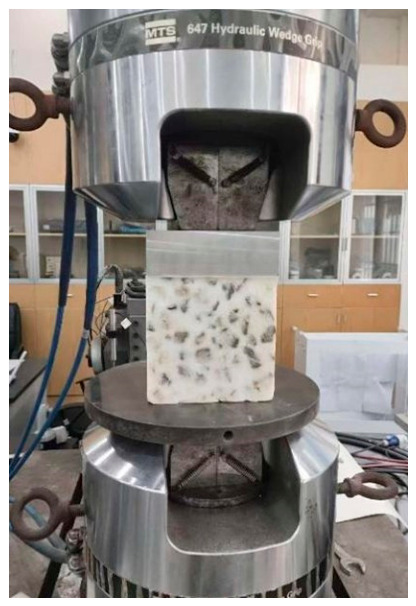
Unconfined test site.

The loading apparatus was the T-slot table test system (MTS 322) from the Institute for Advanced Study at Central South University. The test site is illustrated in Figure 4.

#### 2.2.2. Confined Tests

**Figure 5:**
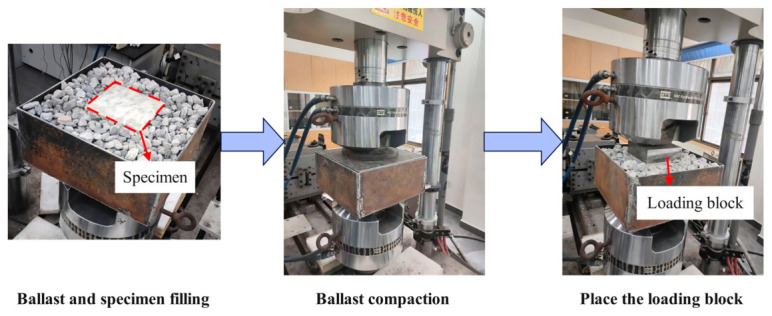
Testing of the PSB specimens under confined conditions.

To simulate the interaction between bulk ballast and the PSB, the ballast box compression test is customized. It can reflect the stress state of the PSB modules under actual service conditions. The PSB specimens prepared from the same batch are placed into a box filled with bulk ballast. The box dimensions were 350 × 350 mm, which are more than twice the specimen’s edge length. This provides approximately 100 mm of surrounding bulk ballast on each side of the specimen and was intended to reduce lateral boundary effects. Under this configuration, the surrounding ballast can deform and redistribute stress before the specimen response is directly influenced by the rigid box wall. After the PSB specimen was placed in the box, the surrounding ballast was filled and compacted in layers. Following box preparation, pre-compaction was conducted using cyclic loading, $\sigma_{min}$ = 18.37 kPa and $\sigma_{max}$ = 36.73 kPa, at a frequency of 4 Hz for 1000 cycles. A loading block was positioned for formal loading after compaction. The test setup is illustrated in Figure 5.

The confined test considers three loading conditions:

(1) Load–unload test: The load amplitude was the same as that of the unconfined test (10 kN), with a strain rate of 1.33%/min. The confined tests were also conducted three times. The specimens were designated as W1, W2, and W3, respectively. The stress–strain data under a single loading–unloading cycle were recorded.

(2) Monotonic compression test: After W1, W2, and W3 specimens had recovered from deformation, the three PSB specimens were compressed to a 30% vertical strain at the same loading rate of 1.33%/min. Stress–strain data and post-test deformation were recorded for comparison with the unconfined test.

(3) Cyclic loading test: Three additional specimens of the PSB from the same batch were selected and designated as P1, P2, and P3, respectively. In order to study the mechanical response of the PSB during the short-term small deformation stage, cyclic loading was applied to the specimens at a frequency of 3 Hz, with 15,000 cycles. The stress–strain data and displacement changes were recorded.

**Figure 6:**
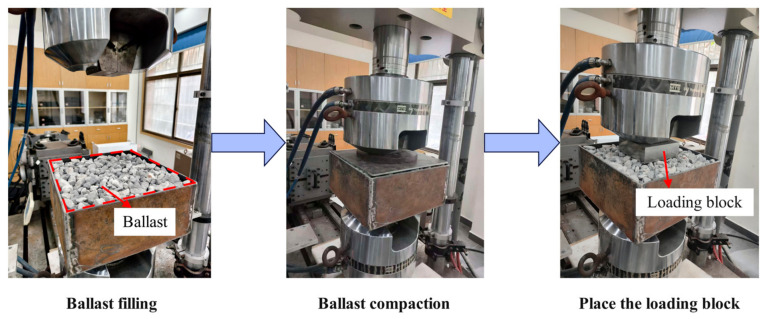
Testing of the pure ballast in a ballast box.

For comparison with traditional ballasted beds, a test box containing pure ballast was also prepared and subjected to tests identical to loading conditions (1) and (3), as shown in Figure 6.

## 3. Experimental Results

### 3.1. The Energy Dissipation Characteristics Under the Load–Unload Condition

**Figure 7:**
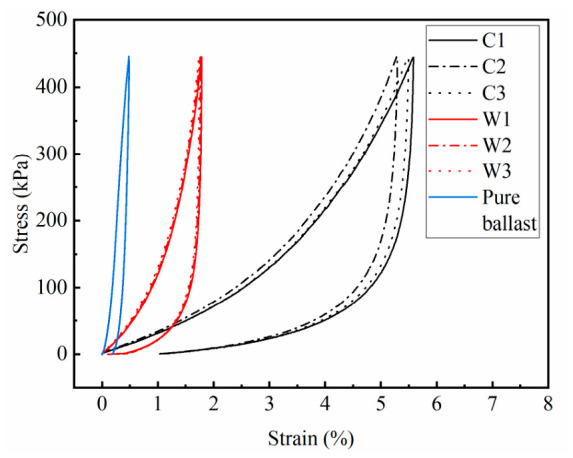
The stress–strain curves of the unconfined (C) and confined (W) PSB specimens and the pure ballast during loading–unloading tests.

The loading–unloading deformation curves of the PSB specimens under unconfined and confined conditions, alongside the pure ballast specimen, are shown in Figure 7. The graph indicates that, during the loading stage, the stiffness of the PSB specimens under both conditions increases significantly with rising strain, whereas the stiffness of the pure ballast exhibits minimal variation. Upon reaching peak load, the maximum strain for the pure ballast was 0.48%. This is because, after preloading, the pure ballast has reached a compacted state and formed a stable stress-bearing structure. The subsequent loading did not alter this system, resulting in minimal stiffness variation. The increase in strain was mainly due to the further compression of the voids between ballast particles. The maximum strains for the three PSB specimens in the confined group were 1.78%, 1.76%, and 1.73%, with an average of 1.76% and a standard deviation of 0.025%. In contrast, the three specimens in the unconfined group exhibited maximum strains of 5.87%, 5.49%, and 5.58%, with an average of 5.65% and a standard deviation of 0.199%. During the loading of the PSB specimens, the polyurethane foam cells were compacted. The contribution of polyurethane to the specimen’s deformation gradually diminished, while the aggregate framework exhibited greater resistance to the deformation. Consequently, the overall stiffness of the specimens increased. As for the unconfined group, lateral deformation was unconstrained, leading to faster strain growth and greater overall deformation compared to the confined group.

The area of the hysteresis loop can effectively characterize the energy dissipation characteristics of specimens during the loading–unloading process. A larger hysteresis loop area indicates greater mechanical energy absorbed and converted by the specimen during cyclic deformation, reflecting a superior energy dissipation capacity. The results reveal that the hysteresis loop area for the three specimens in the unconfined group was 589.19 kJ/m^3^, 557.82 kJ/m^3^, and 568.44 kJ/m^3^, respectively, with an average of 571.82 kJ/m^3^ and a standard deviation of 15.955 kJ/m^3^; the confined group were 175.03 kJ/m^3^, 175.17 kJ/m^3^, and 177.40 kJ/m^3^, respectively, with an average of 175.87 kJ/m^3^ and a standard deviation of 1.330 kJ/m^3^. Both groups were much larger than those of the pure ballast (60.91 kJ/m^3^). This disparity arises from the unique cellular structure within polyurethane, which effectively buffers external loads. Furthermore, the lateral deformation of the unconfined specimens was unrestricted, unlike the confined specimens, where partial energy was absorbed by the confining ballast.

### 3.2. The Stiffness Evolution Under Monotonic Compression

**Figure 8:**
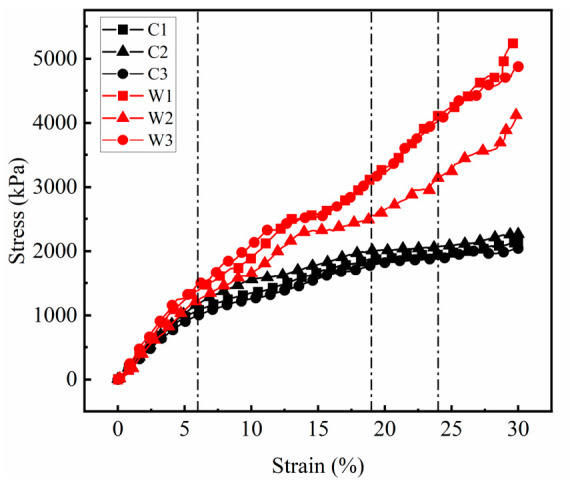
The stress–strain curves of the unconfined (C) and confined (W) PSB specimens during unconfined and confined compression tests.

The monotonic compression stress–strain curves for the PSB specimens are shown in Figure 8. Under unconfined conditions, all three specimens exhibit similar stiffness evolution trends. Based on stress–strain evolution characteristics, the loading process of specimens under unconfined conditions can be divided into three stages:

(1) Linear elastic stage: Stress increases linearly with the strain, reaching a maximum strain of approximately 6%. Specimens undergo vertical compression and lateral expansion without surface failure. Deformation primarily results from the compaction of the polyurethane foam cells and the aggregate, with the aggregate framework bearing the main load.

(2) Stiffness decay stage: Stress growth rate gradually diminishes, with maximum strain reaching approximately 19%. Lateral deformation continues to increase, surface bulging occurs, and surface ballast is extruded. The restraining effect of polyurethane and the interlocking friction between aggregate particles fail to suppress lateral aggregate movement, causing the surface polyurethane to reach its tensile limit and crack.

(3) Crack propagation stage: Stress growth rate markedly slows, with maximum strain reaching approximately 24%. Existing cracks further extend, exposing more aggregate particles, and specimen lateral deformation becomes pronounced.

Further meso-level analyses will be presented in Section 4 in conjunction with the results of the DEM simulation.

During the linear elastic stage, stress increases linearly with strain. The elastic modulus E of the PSB specimens can be calculated from the stress–strain relationship in this stage using Equation (1). Taking the stress increment $∆\sigma_{y}$ and strain increment $∆\varepsilon_{y}$ for vertical strains ranging from 2% to 4%, the calculated elastic modulus of the specimen is presented in Table 3.

(1) $$E=\frac{∆\sigma_{y}}{∆\varepsilon_{y}}$$

The stress–strain curves of the ballast box compression tests of the PSB specimens are shown in Figure 8. Unlike the unconfined condition, when subjected to the confining pressure of bulk ballast, the stress–strain curves of the specimens do not exhibit a distinct three-stage progression but rather display an approximately linear relationship. During the initial linear elastic stage, where strain values are below 6%, stress increases linearly with strain for both groups of specimens. At equivalent strain values, the stress values of the confined group are slightly higher than those of the unconfined group. Subsequently, stress continues to increase with strain. The stiffness of the confined specimens shows minimal change, whereas the unconfined specimens exhibit significant decay. This occurs because the confining ballast restricts the lateral deformation of the specimens. The localized polyurethane within the specimens has not yet reached its strength limit and can still bear loads in conjunction with the aggregate framework. Evidence supporting this view is shown in Table 3, where the average compression modulus of the PSB specimens under confining pressure was 26.26 MPa—a 33.57% increase compared to the elastic modulus measured in the unconfined group. This indicates that the PSB specimens exhibit superior load-bearing capacity when placed in the bulk ballast bed, which provides confinement. The confining pressure exerted by the ballast significantly enhances the deformation resistance of the PSB specimens.

### 3.3. The Dynamic Characteristics Under Cyclic Loading

**Figure 9:**
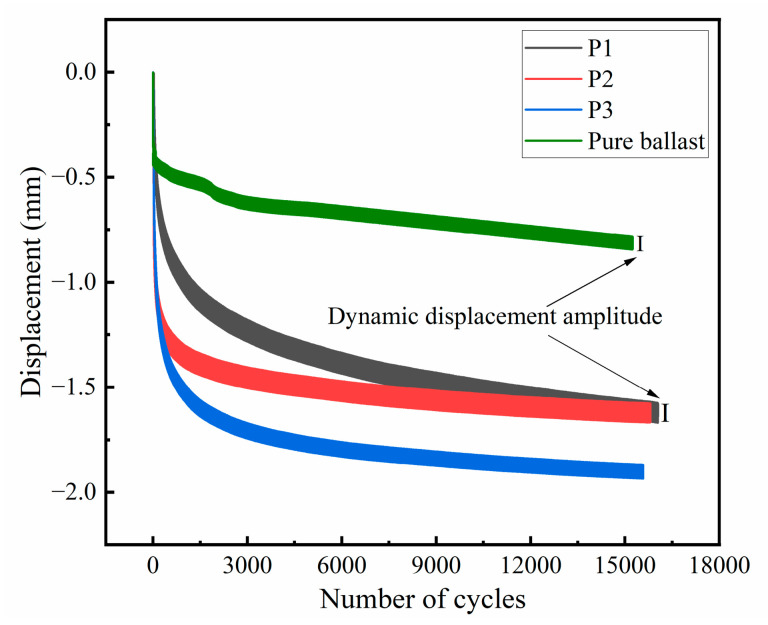
The displacement of the PSB specimens and the pure ballast during cyclic loading tests.

The displacement of the PSB specimens and the pure ballast specimen under cyclic loading is shown in Figure 9. It can be observed that, within 15,000 cycles, the displacements of both specimen groups continue to increase as the number of cycles increases. During the initial stage (less than 3000 cycles), both specimens exhibit rapid displacement changes. Subsequently, the rate of displacement change for the PSB specimens gradually slows, whereas that for the pure ballast remains virtually unchanged. The maximum deformation of the three PSB specimens was 1.89 mm, which was greater than that of the pure ballast (0.84 mm). The dynamic displacement amplitude of the PSB also surpassed that of the pure ballast. This indicates that the support stiffness of PSTB is lower than that of traditional ballasted beds, a finding corroborated by both field tests [40] and discrete element simulations [17].

**Figure 10:**
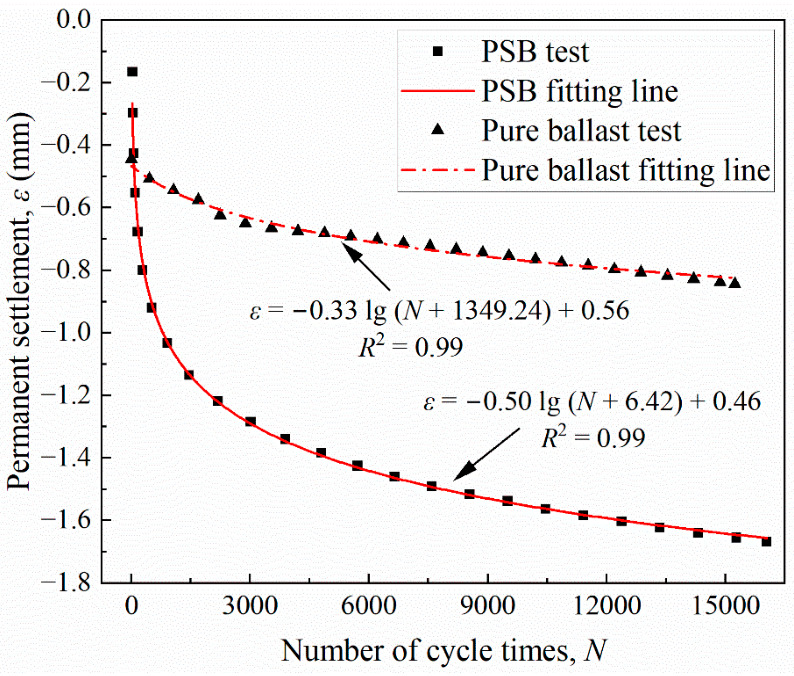
Permanent settlement variation with the number of load cycles.

The permanent settlement values of the PSB and the pure ballast specimen that changed with the number of load cycles are shown in Figure 10. The variation laws can be fitted using a logarithmic function (see Equation (2)), with $R^{2}$ values of 0.99 for both.

(2) $$\varepsilon =-\alpha \log\left(N+\beta \right)+\gamma$$

Parameter α characterizes the deformation sensitivity of the specimen to cyclic loading and the rate of cumulative deformation development; Parameter β reflects the specimen’s initial structural state and equivalent preloading effect; and Parameter γ corresponds to a fitted parameter associated with the later-stage settlement level within the empirical model.

Compared to traditional ballast, the PSB exhibits a larger α and a smaller γ. This is because, in the initial stage of the loading cycle, polyurethane materials primarily exhibit viscous flow and accumulative micro-damage. This necessitates the undertaking of additional cycles to achieve initial stability. However, effective deformation becomes progressively constrained in the later stages. This demonstrates the typical deformation evolution characteristic of the PSB, which is rapid initial development followed by gradual stabilization later. It should be noted that, after the initial rapid settlement stage, the apparent settlement rates of the PSB and pure ballast become closer. This is mainly because the ballast box test reflects the coupled response of the specimen and the surrounding bulk ballast. For the PSB, the initial deformation is strongly affected by polyurethane compaction and local bonded-structure adjustment, whereas the later-stage deformation is increasingly governed by the confinement and rearrangement of the surrounding ballast. Therefore, the convergence in the later settlement rate is mainly considered to be related to the presence of the bulk ballast rather than indicating identical deformation mechanisms in the two systems. In contrast, the pure ballast primarily undergoes particle rearrangement and friction slip, stabilizing quickly in the initial stage but exhibiting more pronounced long-term deformation.

**Figure 11:**
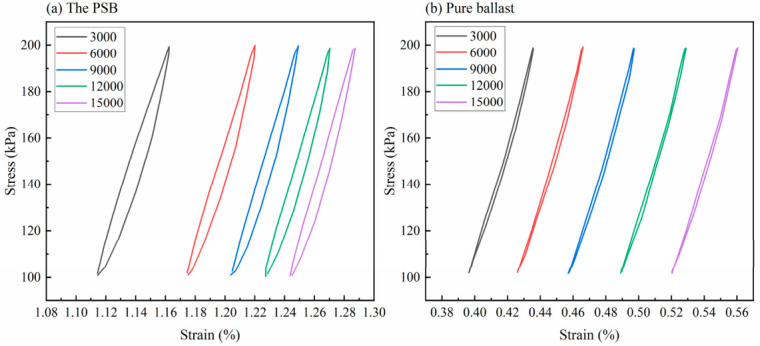
The stress–strain curves of the PSB specimens and the pure ballast during cyclic loading tests.

**Figure 12:**
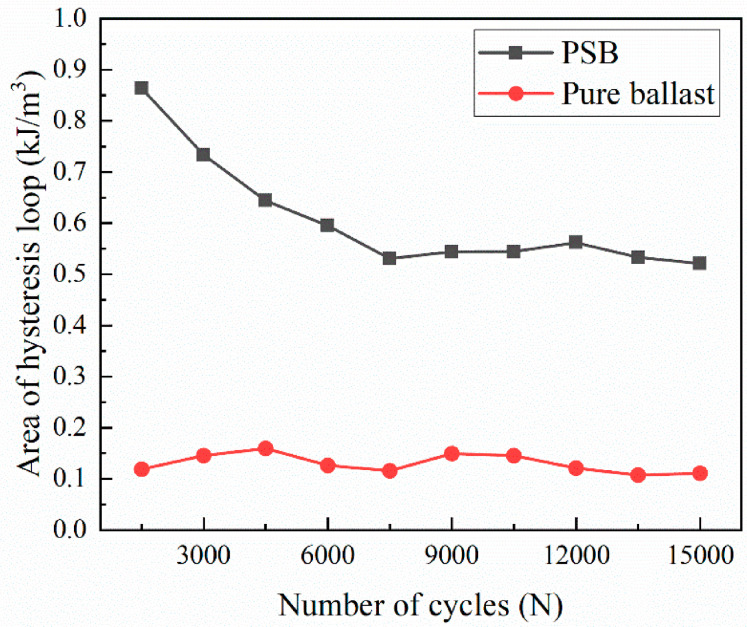
The area of the hysteresis loop variation with the number of load cycles.

The stress–strain curves under cyclic loading were recorded every 3000 cycles, with results shown in Figure 11. The strain growth rate of the PSB specimens gradually slowed with the increasing cycle count, whereas that of the pure ballast specimen exhibited near-linear growth with increasing cycles. The variation in the hysteresis loop area for both PSB specimens and the pure ballast specimen at each cycle count is illustrated in Figure 12. As the number of cycles increased, the hysteresis loop area for the PSB specimens gradually decreased, whereas that for the pure ballast remained largely consistent. Under each cycle of loading, the hysteresis loop area of the PSB specimens is always larger than that of the pure ballast specimen, further confirming that the energy dissipation capacity of the PSB specimens is superior to that of the pure ballast.

**Figure 13:**
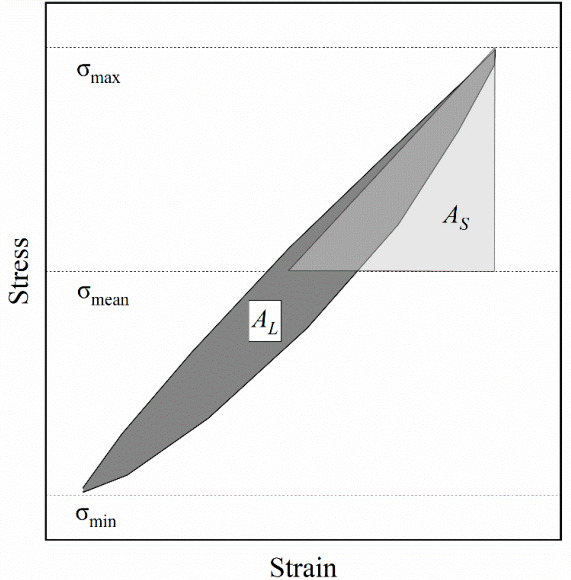
Schematic diagram of damping ratio calculation.

The damping ratio is defined as the ratio of energy dissipated to energy stored during a loading–unloading cycle, reflecting the energy loss within the system under cyclic loading. Its calculation formula is:

(3) $$D=\frac{A_{L}}{4\pi A_{S}}$$

where D is the damping ratio, $A_{L}$ is the area of the hysteresis loop, and $A_{S}$ is the area of the shaded triangle. The calculation schematic diagram is shown in Figure 13.

**Figure 14:**
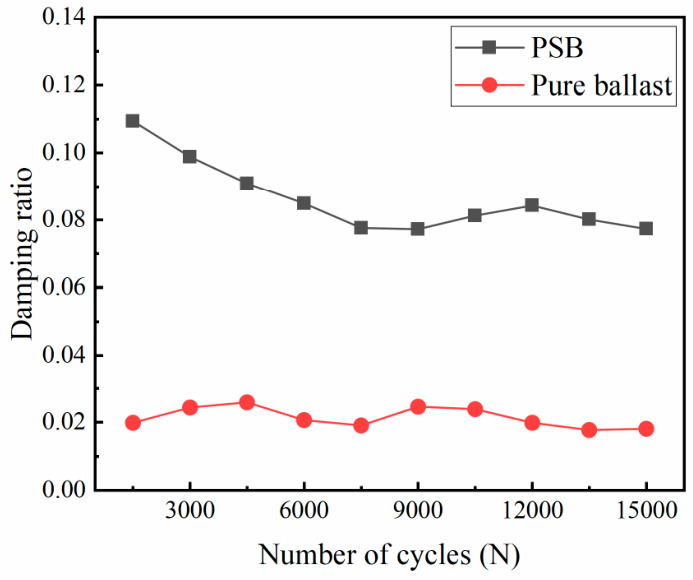
The damping ratio variation with the number of load cycles.

The variation laws of the damping ratio of the PSB and the pure ballast (calculated according to Equation (3)) with the number of load cycles are shown in Figure 14. The figure demonstrates that the damping ratio of the PSB specimens in each cycle is significantly higher than that of the pure ballast specimen. The damping ratio of the PSB specimens decreases with increasing load cycles, whereas that of the pure ballast remains largely unchanged. This discrepancy reflects the fundamental differences in the energy dissipation mechanisms and their evolution with cycling between the two systems. For the PSB specimens, the reduction in the damping ratio with loading cycles is directly observed at the macroscopic level. To explain this trend, a microstructural interpretation is proposed: in the early stage, the damping of the PSB is likely related to the viscoelastic hysteresis of polyurethane and the irreversible adjustment at bonded interfaces; with continued cycling, local damage accumulation may reduce the hysteretic contribution, resulting in a lower damping ratio. The subsequent DEM results provide partial support from the viewpoint of bond degradation, which will be analyzed in Section 4.

In contrast, in the pure ballast system, there is no polymer viscoelastic phase. The dissipation of energy at the macroscopic level is predominantly contributed to by the contact friction and rolling resistance between the aggregates and limited particle rearrangement during the initial stage. Within a few loading cycles, the inter-particle contact network and force chain structure rapidly undergo “self-compaction” and rearrangement. Thereafter, the system stabilizes, with its coefficient of friction, normal constraint, and contact patterns remaining basically unchanged with respect to the load cycles under the given confining pressure and load levels. Consequently, the area of the hysteresis loop and the energy dissipated per unit volume per cycle remain approximately constant, and the corresponding damping ratio shows little variation with the cycle count.

From an engineering perspective, polyurethane curing can significantly enhance the energy dissipation capacity and vibration-damping performance of the ballast layer during the early stages. However, its damping ratio exhibits cyclic decay, implying that vibration-damping effectiveness may gradually diminish over long-term service life. Consequently, the evolution of viscoelastic-damage characteristics in polyurethane bonding materials must be considered in both the design and maintenance processes, as it influences track dynamic response and fatigue safety.

### 3.4. The Shape of the PSB Specimens After Loading

**Figure 15:**
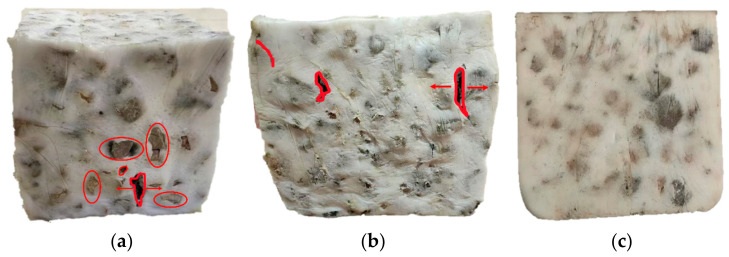
The shape of the PSB specimens after testing: (**a**) monotonic compression test; (**b**) ballast box compression test; and (**c**) cyclic loading test.

The damage pattern of the specimen after the unconfined monotonic compression test is shown in Figure 15a. The figure reveals that, under axial loading, the surface ballast particles underwent misalignment and slip, causing tearing at the adhesive interface between the particles and polyurethane, and the lower part of the ballast particles in the specimen bulged significantly, specifically manifested as the following: the polyurethane encapsulation layer underwent tensile and shear failure during particle displacement, forming strip-like cracks distributed along the particle surfaces.

The final shape of the specimen after the confined monotonic compression test is shown in Figure 15b. As loading progressed, the upper confining ballast was gradually lifted due to lateral deformation of the specimen, progressively reducing the confining pressure on the upper portion. Consequently, the specimen ultimately exhibited a larger upper section and a smaller lower section. No through cracks were observed in the specimen nor was there significant ballast protrusion, though the surface displayed distinct polyurethane wear marks.

The final form of the specimen after the cyclic loading test is shown in Figure 15c. After 15,000 cycles, the specimen retained its integrity with only minor surface wrinkling. This indicates that the specimen maintained its integrity within the tested cycle range. Utilizing the PSB in practical engineering applications can help reduce the cumulative settlement of the track bed under repeated train loads.

## 4. DEM Modeling and Mesoscopic Analysis

### 4.1. DEM Mesoscopic Model

This section aims to establish DEM models for the monotonic compression of the PSB under both unconfined and confined conditions and to elucidate the mesoscopic stiffness evolution laws under these two conditions from the particle scale.

#### 4.1.1. Modeling Procedure

**Figure 16:**
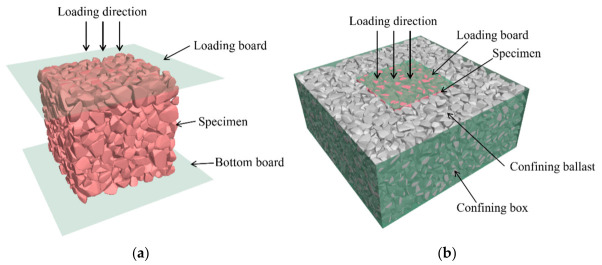
DEM models of laboratory tests: (**a**) monotonic compression model; (**b**) ballast box compression model.

The DEM discretizes materials into non-deformable rigid elements. This study employs Rigid Block elements within the Particle Flow Code 3D (PFC^3D^) platform to simulate ballast particles. Ballast particles are modeled by polygon elements called Rigid Blocks in PFC^3D^. There are six refined particle templates and 120–250 facets in each template to capture the real angular shape of fresh ballast. The particle templates and generation method are detailed in the previous study [33]. The DEM model for the monotonic compression test is illustrated in Figure 16a, comprising three primary components: the specimen (a cube with a side length of 150 mm), a loading plate, and a base plate. The DEM model for the ballast box compression test is depicted in Figure 16b. The model comprises a confining pressure box, confining ballast, the specimen, and a loading plate. The confining pressure box measures 350 × 350 × 150 mm, while the loading plate dimensions match those of the specimen. During the loading stage of the test, the loading plate compresses the specimen at a constant rate, and the displacement and contact forces of the model are recorded.

#### 4.1.2. Model Validation

This section presents the parameter selection and model validation for the DEM model. The adopted parameter ranges and preliminary values were established based on the authors’ previous study [33], and the experimental results in the present study were used to examine whether the model could reasonably reproduce the observed macroscopic response under the current loading conditions.

**Figure 17:**
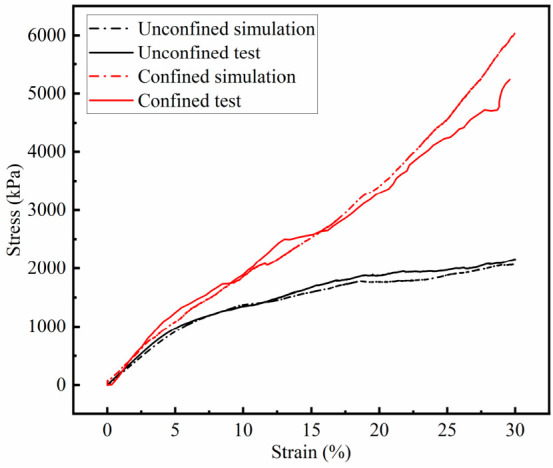
Comparison of test results with numerical simulation results.

The bonded and unbonded contact parameters for the DEM models are presented in Table 4 and Table 5. The comparison of results, as shown in Figure 17, demonstrates good agreement between numerical simulations and laboratory test data, thereby confirming that the selection of relevant parameters is appropriate.

### 4.2. Mesoscopic Analysis of Unconfined Test Simulation

**Figure 18:**
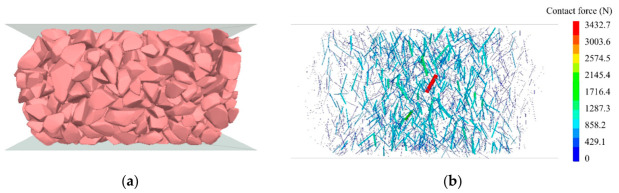
Results of monotonic compression test simulation: (**a**) deformation; (**b**) distribution of contact forces.

Figure 18 illustrates the deformation of the specimen and the distribution of contact forces during the monotonic compression simulation test loading process. The figure reveals that the specimen undergoes vertical compression and lateral expansion under vertical loading, reflecting the restraining effect of polyurethane on specimen deformation. Contact forces were predominantly concentrated in the central region of the specimen, with a maximum contact force of 3432.7 N. Contact forces decreased progressively with the distance from the specimen’s center. This indicates that, after compression, the framework formed by ballast particles in the central region bore the primary external load.

The state of the bonds corresponding to the strain values of 6%, 19%, 24%, and 30%, respectively, during the monotonic compression simulation is statistically analyzed, with the results presented in Table 6. As evident from the table, at the initial stage, all bonds are bonded. With the increasing load, the number of bonded bonds and the coordination number gradually diminish. Before reaching 6% strain, most of the bonds retain their bonded state. A minority of bonds reach their tensile strength limit and soften. The number of softened bonds progressively increases with the strain, peaking at the end of the linear elastic stage. Upon entering the stiffness decay stage, there is an observable increase in the number of broken bonds that exceed their strength limit. Concurrently, there is a significant decrease in both the number of bonded bonds and the coordination number. New rigid contacts form between some ballast particles. At a strain value of 19%, the number of bonds in a softened compressed state reaches its peak. Upon entering the crack propagation stage, the number of broken bonds continues to increase with specimen compression. At a strain value of 24%, the number of broken bonds approaches its peak. When the strain reaches 30%, the number of rigidly bonded contacts peaks, indicating that, after compression, a substantial number of ballast particles no longer maintain a bonded connection but instead rely on rigid contact between the ballast particles.

### 4.3. Mesoscopic Analysis of Confined Test Simulation

**Figure 19:**
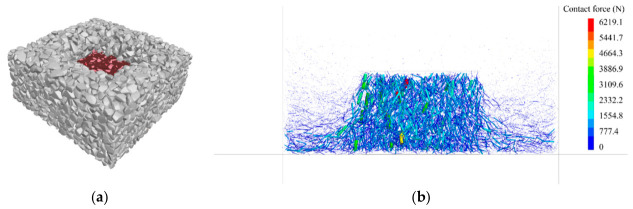
Results of ballast box compression test simulation: (**a**) deformation; (**b**) distribution of contact force.

The deformation of the specimen and the distribution of contact forces during the simulation loading process of the ballast box compression test are shown in Figure 19. It can be seen from the figure that the lateral deformation caused by the compressed specimen makes the surrounding ballast bulge upwards. Significant contact forces were recorded within the specimen, with a maximum value of 6219.1 N. The surrounding ballast exhibited lower contact forces and a more dispersed distribution compared to the specimen. The ballast box compression test demonstrated a more uniform distribution of contact forces within the specimen compared to monotonic compression tests. This is attributed to the confining ballast restricting the lateral deformation of the specimen, thereby enabling the entire specimen to collectively bear the external load.

The state of the bonds corresponding to the strain values of 6%, 19%, 24%, and 30%, respectively, during the ballast box compression simulation is statistically analyzed, with the results presented in Table 7. As shown in the table, under confined conditions, the number of bonded bonds and the coordination number also gradually decrease with the increasing load, but the reduction is smaller than that of unconfined conditions. Unlike in monotonic compression, where the number of bonded bonds and the coordination number drop significantly once the specimen enters the stiffness decay stage, this microstructural phenomenon clearly demonstrates the reinforcing effect exerted by the confining pressure within the bulk ballast. Upon the completion of loading—when the strain reached 30%—both the number of broken bonds and the quantity of newly formed rigid contacts are lower than those of unconfined conditions, while the coordination number is higher. This indicates that greater polyurethane bonding action persists within the PSB. Consequently, the specimen under confined conditions retains higher stiffness even at a 30% strain.

**Figure 20:**
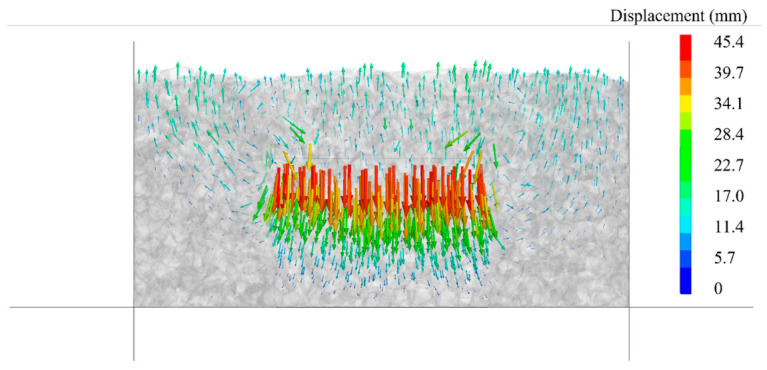
Particle displacement variation in ballast box compression test simulation.

The displacement variation in the aggregate particles during the ballast box compression test is illustrated in Figure 20. In this test, the specimen exhibited a maximum compressive displacement of 45.4 mm. The lateral deformation induced by the compressed specimen caused the surrounding ballast to heave upwards, with the uplift height measuring approximately 15 mm.

### 4.4. Mesoscopic Comparison Under Unconfined and Confined Conditions

**Figure 21:**
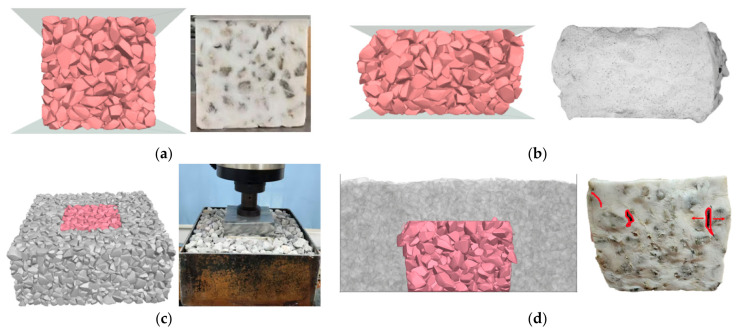
Comparison of monotonic compression and ballast box compression test and simulation before and after loading: (**a**) before monotonic compression; (**b**) after monotonic compression; (**c**) before ballast box compression; and (**d**) after ballast box compression.

Through comparative analysis of post-loading deformation patterns in Figure 21, it is evident that the numerical simulation results accurately reproduce the experimental phenomena. The deformation characteristics of specimens in both simulation groups align with experimental observations: the unconfined test exhibits bulging at both ends, whereas the confined test primarily shows pronounced lateral expansion in the upper half of the specimen, with relatively minor deformation at the base.

**Figure 22:**
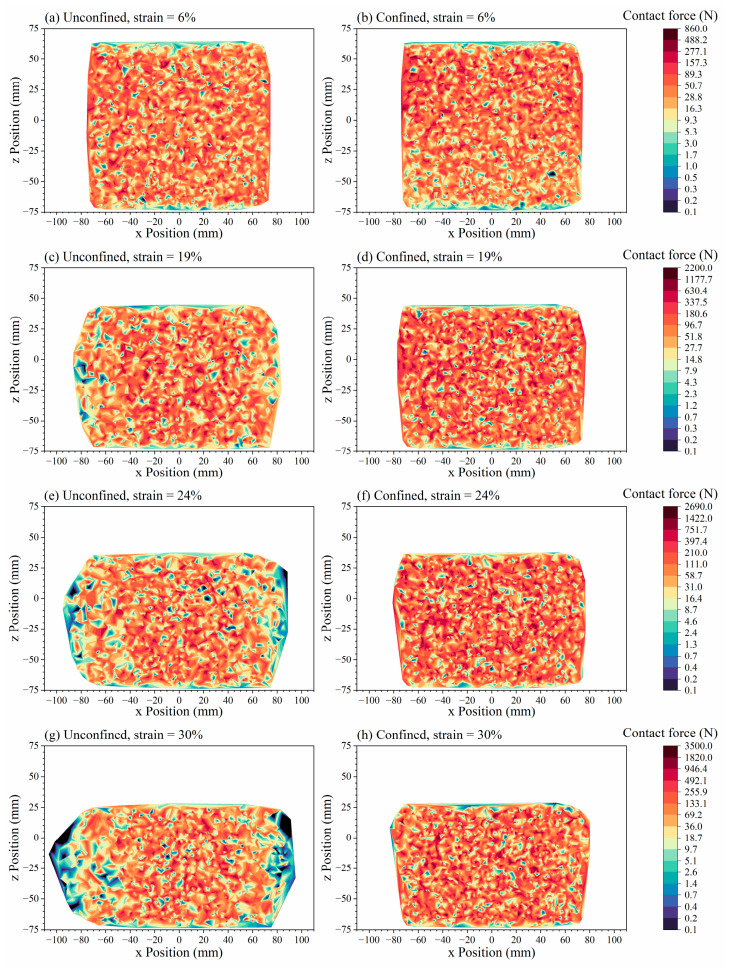
Contact forces contour plots of the PSB specimens.

Figure 22 presents the contact forces contour plots for monotonic compression and ballast box compression simulations at strain values of 6%, 19%, 24%, and 30%, respectively. The figure reveals that, at equivalent strain levels, specimens with lateral confinement exhibit a more uniform distribution of contact forces and reduced lateral deformation, with maximum internal contact forces exceeding those in unconfined specimens. This arises because the confining ballast restricts specimen deformation, enabling the entire specimen to collectively bear external loads. The simulation shapes of specimens also align with the deformation patterns observed in the actual testing.

## 5. Discussion

The test results show that the behavior of PSB is strongly affected by the surrounding ballast. Under unconfined compression, the PSB shows a clear three-stage response, including elastic deformation, stiffness reduction, and crack development. Under confined conditions, the response becomes more linear and the stiffness increases. This means that PSB should not be treated as an isolated block. In practice, it works together with the surrounding ballast.

A key result of this study is that confinement clearly improves the mechanical performance of PSB. The confined specimens showed a 33.57% higher modulus than the unconfined specimens. This is important for engineering use, because PSB modules in track beds are always surrounded by bulk ballast. The surrounding ballast limits lateral deformation and helps the bonded aggregate skeleton carry more load. The DEM results support this point. Under confined conditions, the contact forces inside the specimen are more uniform, and the bonded contacts are better preserved during loading. This explains why the confined specimens keep a higher stiffness. From an engineering point of view, this means that the PSB can provide higher deformation resistance and more stable support when working together with the surrounding ballast.

The cyclic tests also show clear differences between the PSB and pure ballast. PSB has larger deformation, especially in the early stage. This means that PSB is more deformable than pure ballast. However, PSB also shows a much larger hysteresis-loop area and damping ratio. In other words, it can dissipate more energy and provide better vibration reduction. Therefore, the larger deformation of PSB should not be seen as only a disadvantage. It also reflects a different balance between stiffness and damping.

The settlement curves further show that PSB and pure ballast have different evolution patterns. PSB shows fast initial settlement, but the growth rate decreases later. Pure ballast shows a more continuous increase. This suggests that PSB experiences an early adjustment stage, and then gradually becomes more stable. At the same time, the later settlement rates of the two systems become closer. This is likely because the cyclic response is increasingly influenced by the surrounding ballast. Therefore, the ballast box test reflects the combined behavior of PSB and bulk ballast not the PSB alone.

The damping results provide another important finding. The damping ratio of PSB is always much higher than that of pure ballast, but it decreases with loading cycles. This means that PSB has a strong energy dissipation ability at the beginning, but this effect weakens gradually with repeated loading. A possible explanation is that the polyurethane and the bonded interfaces undergo gradual damage during cyclic loading. This explanation is based on the macroscopic test results. The DEM results give partial support, because they show that bonded contacts decrease and broken contacts increase during loading. Still, the current DEM model mainly describes bond softening and breakage, and it cannot fully capture the cyclic viscoelastic behavior of polyurethane.

An important contribution of this study is the combination of experiments and DEM analysis. The DEM model reproduces the main deformation patterns observed in the tests. More importantly, it helps explain the internal mechanisms that cannot be directly observed in the laboratory. The simulations show that, in the unconfined case, the central aggregate framework carries most of the load. In the confined case, the load is distributed more evenly through the specimen. This helps explain why confinement increases stiffness and delays damage development.

This study is subject to several limitations. The tests were performed on scaled laboratory specimens under controlled boundary conditions, and the number of repeated specimens was limited. The cyclic loading program covered only 15,000 cycles, so the results mainly reveal comparative evolution trends rather than full long-term service performance. In addition, the current DEM model mainly describes bond softening and breakage and cannot fully capture the cyclic viscoelastic-damage behavior of polyurethane. Therefore, the present findings should be interpreted within the scope of the tested conditions.

## 6. Conclusions

This study investigated the mechanical behavior of polyurethane-solidified ballast (PSB) under unconfined and confined conditions through laboratory tests and DEM analysis. The main conclusions are as follows.

(1) The mechanical response of PSB is strongly affected by the surrounding ballast. Under unconfined compression, the PSB shows a three-stage response with progressive stiffness reduction and crack development. Under confined conditions, the response becomes more linear, and the stiffness increases significantly. This shows that the surrounding ballast does not only provide confinement but also improves the load-carrying efficiency of the bonded aggregate skeleton.

(2) Compared with pure ballast, PSB shows a different balance between deformation and energy dissipation. Although PSB exhibits larger deformation during cyclic loading, it also shows a much higher hysteresis-loop area and damping ratio. This indicates that PSB has a stronger vibration-reduction and energy-dissipation capacity, which may be beneficial in railway sections requiring both deformation adaptability and impact buffering.

(3) The DEM results help explain experimental observations from a mesoscopic point of view. The simulations reproduce the main deformation patterns and show that confinement leads to a more uniform contact-force distribution and slower bond degradation. This confirms that the interaction between the bonded aggregate skeleton and the surrounding ballast is the key mechanism controlling the macroscopic behavior of PSB.

(4) The present study mainly clarifies the comparative mechanical trends and mesoscopic mechanisms under the tested laboratory conditions. The cyclic test duration and specimen number are still limited, and the current DEM model mainly captures bond softening and breakage rather than the full cyclic viscoelastic evolution of polyurethane.

Based on these findings, future work should focus on longer-duration cyclic tests with a larger number of specimens, the direct observation of interface and polyurethane damage evolution, and the development of improved DEM contact models for cyclic viscoelastic-damage behavior.

## Figures and Tables

**Table 1:** Ballast particle size distribution.

Side Length of Sieve Hole (mm)	26.5	21.5	16	13.2	9.5
Percentage of sieved mass (%)	100	70	35	10	0

**Table 2:** The content of each particle size range of aggregate in a single specimen.

Range of Particle Size (mm)	9.5–13.2	13.2–16	16–21.5	21.5–26.5
Aggregate mass (kg)	0.56	1.39	1.95	1.67

**Table 3:** Elastic modulus and compression modulus of the PSB specimens.

Specimen Label	Elastic Modulus (MPa)	Specimen Label	Compression Modulus (MPa)
C1	19.63	W1	27.50
C2	20.80	W2	25.09
C3	18.57	W3	26.20
Average	19.66	Average	26.26
Standard deviation	1.115	Standard deviation	1.206

**Table 4:** Parameters of soft bond model for PSB.

Parameters	Value
Normal stiffness (MPa/m)	500
Stiffness ratio	3.5
Cohesive (kPa)	110
Tensile strength (kPa)	110
Softening coefficient	5
Tensile strength coefficient	0
Internal friction angle	45°
Friction coefficient	0.4
Damping coefficient	0.2

**Table 5:** Parameters of linear contact model for normal ballast.

Parameters	Value
Normal contact stiffness (MN/m)	10
Stiffness ratio	1.0
Friction coefficient	0.7
Damping coefficient	0.01

**Table 6:** Percentage of different bond states during the monotonic compression test simulation.

Strain (%)	Bonded (%)	Softened (%)	Broken (%)	Newly Contacted (%)	Coordination Number
0	100	0	0	0	8.62
6	82.0	15.8	1.8	0.4	7.74
19	32.3	16.7	40.7	10.3	5.56
24	24.1	13.2	48.6	14.1	5.23
30	18.9	10.9	50.2	20.0	5.10

**Table 7:** Percentage of different bond states during ballast box compression test simulation.

Strain (%)	Bonded (%)	Softened (%)	Broken (%)	Newly Contacted (%)	Coordination Number
0	100	0	0	0	8.62
6	92.2	6.2	1.2	0.4	8.41
19	54.1	14.9	24.9	6.1	7.52
24	44.8	12.7	34.9	7.5	7.25
30	34.5	10.3	46.8	8.3	6.97

## Data Availability

The original contributions presented in this study are included in the article. Further inquiries can be directed to the corresponding author.
